# Label fusion method combining pixel greyscale probability for brain MR segmentation

**DOI:** 10.1038/s41598-019-54527-x

**Published:** 2019-11-29

**Authors:** Monan Wang, Pengcheng Li

**Affiliations:** School of Mechanical & Power Engineering, Harbin University of Science and Technology, Xue Fu Road No. 52, Nangang District, Harbin City, Heilongjiang Province, 150080 People’s Republic of China

**Keywords:** Machine learning, Biomedical engineering

## Abstract

Multi-atlas-based segmentation (MAS) methods have demonstrated superior performance in the field of automatic image segmentation, and label fusion is an important part of MAS methods. In this paper, we propose a label fusion method that incorporates pixel greyscale probability information. The proposed method combines the advantages of label fusion methods based on sparse representation (SRLF) and weighted voting methods using patch similarity weights (PSWV) and introduces pixel greyscale probability information to improve the segmentation accuracy. We apply the proposed method to the segmentation of deep brain tissues in challenging 3D brain MR images from publicly available IBSR datasets, including images of the thalamus, hippocampus, caudate, putamen, pallidum and amygdala. The experimental results show that the proposed method has higher segmentation accuracy and robustness than the related methods. Compared with the state-of-the-art methods, the proposed method obtains the best putamen, pallidum and amygdala segmentation results and hippocampus and caudate segmentation results that are similar to those of the comparison methods.

## Introduction

Magnetic resonance (MR) imaging technology plays an important role in neuroscience research and clinical applications. Doctors often need to identify regions of interest (ROIs) or diseased tissues from multiple brain images and make judgements or develop treatment plans. At present, a large number of brain images are generated every day in hospitals, and it is a burden for doctors to accurately segment the brain structures by manual segmentation. Therefore, an automatic segmentation technique is needed to deal with the routine analysis of clinical brain MR images. Many automatic image segmentation algorithms have been proposed^1,2^, but due to the complexity of deep brain structures, it is still a challenging task to develop a fast and accurate automatic segmentation method. FreeSurfer^3^ and FIRST^4^ are widely used automatic segmentation methods. FreeSurfer uses non-linear registration and an atlas-based segmentation approach. FIRST takes the deformable-model-based active appearance model and puts it in a Bayesian framework. These two methods alleviate the burden of manual segmentation, but their accuracy still needs to be improved.

In the past decade, atlas-based methods have shown superior performance over other automatic brain image segmentation algorithms^5^. The main idea of the atlas-based method is to use the prior knowledge of the atlas to classify the target pixels. Each atlas contains an intensity image and a label image labelled by a doctor. This method registers the target image with the intensity images and then uses the obtained deformation field to propagate the label information to the target image space to achieve the classification of the target pixel. A disadvantage of atlas-based methods is that they are sensitive to noise and differences between images. This problem can be solved by MAS^6^ and atlas selection^7^.

MAS methods mainly include two steps: image registration and label fusion. At present, many registration algorithms have been proposed^8^. The registration performance of an MAS method has a great influence on the segmentation results. Therefore, most MAS methods use a highly accurate non-linear registration method. Alvén *et al*.^9^ proposed an Überatlas registration method for MAS, which effectively shortens the registration time. Alchatzidis *et al*.^10^ presented a method that integrates registration and segmentation fusion in a pairwise MRF framework.

Label fusion is a key step in MAS methods and can reduce the errors caused by registration errors or morphological differences between the target image and the atlas images. Weighted voting (WV) is a widely used label fusion method. This method can effectively improve the segmentation results. The weight value is mainly defined by the similarity between the atlas images and the target image; examples include the absolute value of the intensity difference^11^, the Gaussian function of the intensity difference^12^, and the local mutual information^13^. CoupeK *et al*.^14^ proposed a hippocampal and ventricular segmentation method based on non-local patches. Wang *et al*.^15^ proposed a multi-atlas active contour segmentation method and used a template optimization strategy to reduce registration error. Lin *et al*.^16^ proposed a label fusion method based on registration error and intensity similarity. Tang *et al*.^17^ proposed a low-rank based image recovery method combined with the sparse representation model, which successfully achieved the segmentation of brain tumours. Roy *et al*.^18^ proposed a patch-based MR image tissue classification method. This method uses the sparse dictionary learning method and combines the statistical atlas prior to the sparse dictionary learning. The entire segmentation process does not require deformation registration of the atlas images. Tong *et al*.^19^ combined discriminative dictionary learning and sparse coding techniques to design a fixed dictionary for hippocampus segmentation. Lee *et al*.^20^ proposed a brain tumour image segmentation method using kernel dictionary learning. This method implicitly maps intensity features to high-dimensional features to make classification more accurate. However, this method is time consuming and cannot be widely applied. To address this problem, a linearized-kernel sparse representative classifier has been proposed^21^.

In recent years, learning-based methods have been widely used in MR image segmentation fields, such as the support vector machine (SVM)^22^, random forest (RF)^23^ and convolutional neural network (CNN)^24^ methods. These methods are suitable for the image analysis of specific anatomical structures due to their advantages in terms of efficiency in pairwise registration and feature description. Huo *et al*.^25^ proposed a multi-atlas segmentation method combined with SVM classifiers and super-voxels. This method is not sensitive to pair registration and reduces registration time by using affine transformation. Xu *et al*.^26^ proposed an atlas forest automatic labelling method for brain MR images. They use random forest techniques to encode atlas images into the atlas forest to reduce registration time. Kaisar *et al*.^27^ proposed a convolutional neural network model combining convolution and prior spatial features for sub-cortical brain structure segmentation and trained the network using a restricted sample selection to increase segmentation accuracy.

Brain tissue boundary regions are difficult to segment for both the MAS and learning-based segmentation methods. It is mainly because the pixel values of the tissue boundary regions are very similar and it is difficult to identify whether the pixel points of these regions belong to the target organization. In order to overcome this shortcoming and make full use of the prior information of the atlas, we introduce pixel greyscale probability information into the label fusion method.

In this paper, we propose a label fusion method combining pixel greyscale probability (GP-LF) for brain MR image segmentation. The proposed method is mainly aimed at improving the segmentation results in the tissue boundary region and improving the segmentation accuracy. We use the SRLF method and the PSWV method to obtain the fusion results of target tissues and then introduce pixel greyscale probability information to fuse the fusion results obtained by the above two methods. The pixel greyscale probability is obtained through a large amount of segmentation training.

## Methods

We propose our method based on the patch-based label fusion method of MAS. In this section, we introduce the patch-based label fusion method and describe the proposed method in detail.

### Dataset

We tested the proposed method using the IBSR dataset. The IBSR dataset is a publicly available MR brain image dataset that is provided by the https://www.nitrc.org/projects/ibsr website. It contains data from 18 T1-weighted MR image data and the corresponding label images, which are defined by the Center for Morphometric Analysis at Massachusetts General Hospital. The 18 subject images were obtained using two different MRI scanners: GE (1.5T) and SIEMENS (1.5T). The images are from 14 males and 4 females and have dimension 256 × 256 × 128 and three different resolutions: 0.84 × 0.84 × 1.5 mm^3^, 0.94 × 0.94 × 1.5 mm^3^ and 1 × 1 × 1.5 mm^3^. Additionally, this dataset is part of the Child and Adolescent NeuroDevelopment Initiative^28^ (CANDI) and was provided under the Creative Commons: Attribute license^29^.

### Patch-based label fusion method

The patch-based label fusion method maps the label information to the target image space by registering the target image and the atlas images and then identifies the target tissue pixel by a label fusion algorithm. The detailed process is as follows:

1. Image registration: The image to be segmented is taken as the target image T, which is registered with the intensity images in the atlas. Saving the warped atlas ${A}_{1}({I}_{1},{L}_{1}),{A}_{2}({I}_{2},{L}_{2}),\cdots {A}_{n}({I}_{n},{L}_{n})$, where *I*_*i*_ is the warped intensity image of atlas *A*_*i*_, *L*_*i*_ is the warped label image of atlas *A*_*i*_, and *n* is the number of atlases, is saved.
2. Patch extraction: The patch *Tp*_*xj*_ centring on the pixel *x*_*j*_ of the target image is extracted, and then patches *Ip*_*i*_ and *Lp*_*i*_ are extracted from the *x*_*j*_ position of images *I*_*i*_ and *L*_*i*_, respectively.
3. Label fusion: A label fusion method is used to fuse the extracted patches.
4. Label assignment: The fusion result is binarized to assign a label (background or target organization) to the pixel *x*_*j*_.

The WV method is a widely used label fusion method; its equation is as follows:

1 $$Fv({x}_{j})=\frac{{\sum }_{i=0}^{n}{w}_{i}{l}_{i}}{{\sum }_{i=0}^{n}{w}_{i}}$$

2 $${L}_{T}({x}_{j})=\{\begin{array}{cc}1, & {\rm{if}}\,Fv(x)\ge {\rm{Binarization}}\,{\rm{threshold}}\\ 0 & {\rm{if}}\,Fv(x) < {\rm{Binarization}}\,{\rm{threshold}}\end{array}\,\,j=1,2\cdots m$$

where *w*_*i*_ is the weight coefficient, *Fv*(*x*_*j*_) is the fusion result, and *m* is the number of target image pixels. If *L*_*T*_(*x*_*j*_) = 0, the pixel *x*_*j*_ is marked as the background, and if *L*_*T*_(*x*_*j*_) = 1, the pixel *x*_*j*_ is marked as a target organization pixel.

The calculation of *w*_*i*_ is the core of the WV method. There are two common methods for calculating label weight: one is to calculate the similarity of $T{p}_{xj}$ and *Ip*_*i*_ and take that as the weight *w*_*i*_, and the other is to calculate the weight *w*_*i*_ by using the sparse representation method.

The SRLF method assumes that *Tp*_*xj*_, *Ip*_*i*_ and *Lp*_*i*_ are one-dimensional column vector signals and uses all the images *Ip*_*i*_ to build an over-complete dictionary *D*_*I*_; the *Tp*_*xj*_ approximately lie in the subspace spanned by the training patches in the library *D*_*I*_.

In this paper, we use $PT{[p{t}_{1},p{t}_{2}\cdots p{t}_{len}]}^{T}$ to represent *Tp*_*xj*_, where *pt*_*i*_ corresponds to each pixel in the patch *Tp*_*xj*_, *len* is equal to the number of pixels in the *Tp*_*xj*_. We use the column vector $P{I}_{i}{[p{i}_{1i},p{i}_{2i}\cdots p{i}_{leni}]}^{T},i=1,2,\cdots m$ to represent *Ip*_*i*_, and the over-complete dictionary *D*_*I*_ is represented as ${D}_{I}[P{I}_{1},P{I}_{2}\cdots P{I}_{m}]$. The sparse coefficient $\alpha ={[{\alpha }_{1},{\alpha }_{2}\cdots {\alpha }_{m}]}^{T}\in Rn$. Each element of the *PT* can be represented by the following formula:

3 $$p{t}_{j}=p{i}_{j1}{\alpha }_{1}+p{i}_{j2}{\alpha }_{2}+\cdots p{i}_{jm}{\alpha }_{m}$$

4 $$PT=D{}_{I}\alpha $$

The idea of sparse representation is to represent the signal *PT* by solving the most sparse solution α. This solution for α can be obtained by solving the following equation:

5 $$\hat{\alpha }=\text{arg}\,{\rm{\min }}\,{\Vert \alpha \Vert }_{0}\,{\rm{subject}}\,{\rm{to}}\,{\Vert P{T}_{i}-D\alpha \Vert }_{2}^{2}\le \varepsilon $$

The solution for α is used as the weight *w*_*i*_ of WV, and then the label fusion is performed by using Eq. (1).

### Proposed method

Equation (5) is an underdetermined equation, such that the number of non-zero coefficients in the sparse solution is equal to the number of elements of the vector *Tp*_*xj*_. Therefore, in the SRLF method, only a small number of patches participate in the fusion process, and most of the patches are discarded, which will lead to segmentation errors. The PSWV method combines all the patches, but is not as good at edge pixel recognition as the SRLF method.

**Figure 1:**
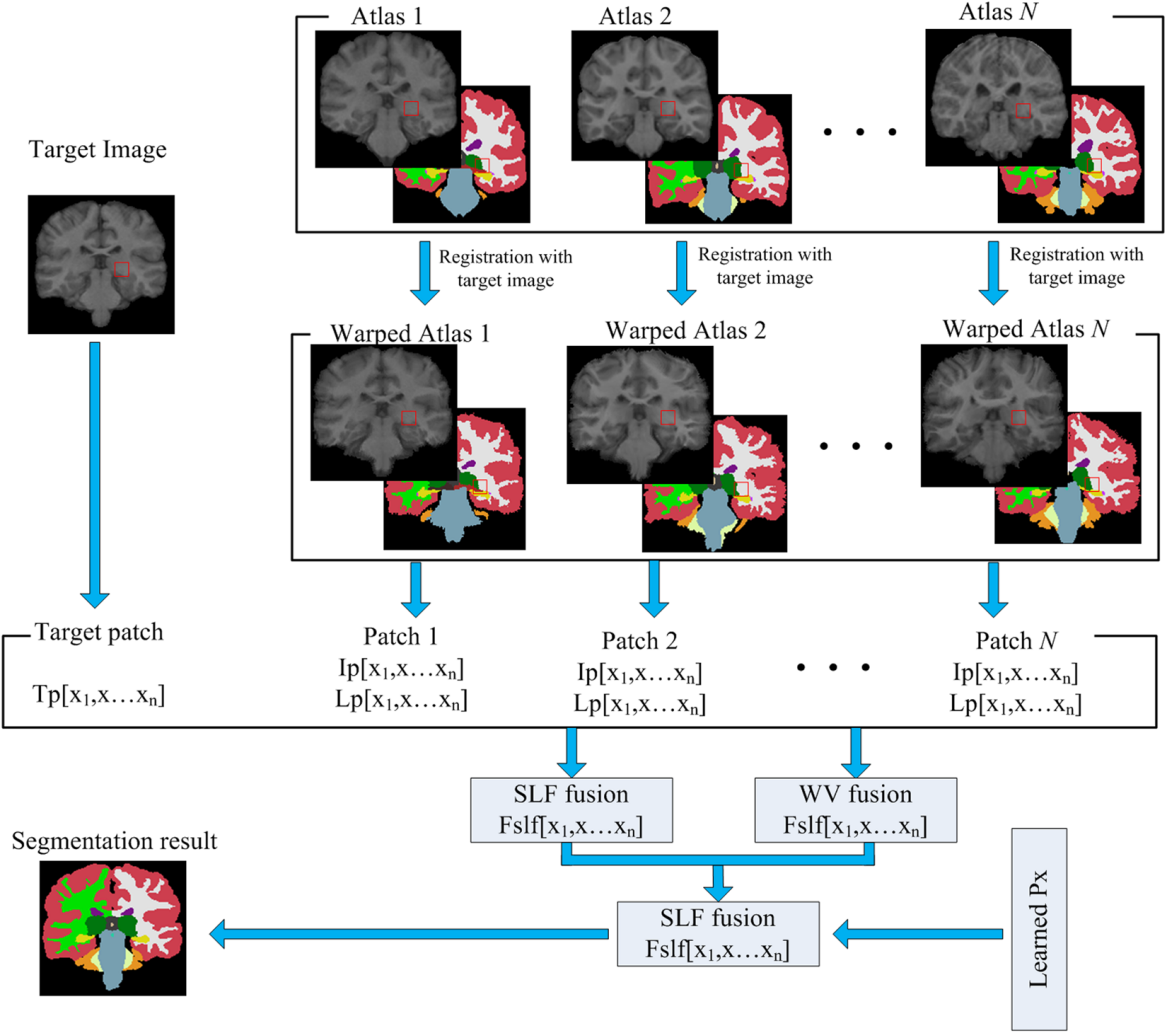
Schematic diagram of the proposed method.

In order to overcome the shortcomings of the above methods, we propose the GP-LF method. The overall framework of the proposed method is shown in Fig. 1. In order to make better use of the prior information of the atlas, we introduce the image grey probability information in the label fusion process and obtain the pixel greyscale probability value *P*(*x*_*j*_) of the target image through segmentation training.

1. Multi-atlas registration: The current non-linear registration method has been able to achieve good registration results, so we used an existing non-linear registration method for multi-atlas registration. We selected the SuperElastix registration tool and used the affine and B-spline deformation methods to register the target image and the atlas intensity images. The SuperElastix registration tool is available at https://github.com/SuperElastix/.
2. Initial label fusion: In order to avoid the influence of non-standard images on the segmentation results, we perform pixel value normalization on the target image and on the atlas intensity images. After multi-atlas registration, the obtained warped atlas is fused using the SRLF method, and the fusion result is represented by *Fv*_*SRLF*_(*x*_*j*_). We use the normalized correlation coefficient (NCC)^30^ as a similar measure function to perform PSWV fusion on the warped atlas images, and the fusion result is represented by *Fv*_*PSWV*_(*x*_*j*_).
3. Establishment of the GP-LF model: The pixel greyscale probability information is introduced to fuse the results *Fv*_*SRLF*_(*x*_*j*_) and *Fv*_*PSWV*_(*x*_*j*_) obtained in the above steps. The fusion equation is as follows:
  6 $${L}_{T}({x}_{j})=\{\begin{array}{cc}1 & {\rm{if}}\,F{v}_{SRLF}({x}_{j}) > 0.9\,{\rm{or}}\,F{v}_{PSWV}({x}_{j}) > {\rm{0.9}}\\ L{f}_{1}({x}_{j}) & {\rm{if}}\,0.4 < F{v}_{SRLF}({x}_{j})\le {\rm{0.9}}\,{\rm{and}}\,{\rm{0.4}} < F{v}_{PSWV}({x}_{j}) < 0.9\\ L{f}_{2}({x}_{j}) & {\rm{if}}\,F{v}_{SRLF}({x}_{j})\le 0.4\\ L{f}_{3}({x}_{j}) & {\rm{if}}\,F{v}_{PSWV}({x}_{j})\le 0.4\end{array}$$
  where *L*_*T*_(*x*_*j*_) is a piecewise function. When *Fv*_*SRLF*_(*x*_*j*_) and *Fv*_*PSWV*_(*x*_*j*_) are greater than 0.9, almost all the identified pixels are target organization pixels. In order to reduce calculation time and not affect segmentation precision, we mark these pixels directly as target organization pixels. When *Fv*_*SRLF*_(*x*_*j*_) and *Fv*_*PSWV*_(*x*_*j*_) are between 0.4 and 0.9, the fusion is performed by using the formula for *Lf*_1_(*x*_*j*_), which is as follows:
  7 $$L{f}_{1}({x}_{j})=\{\begin{array}{cc}1 & fusion1 > 0.5\\ 0 & else\end{array}\,fusion1={{\rm{\beta }}}_{1}\cdot F{v}_{SLRLF}({x}_{j})\cdot F{{v}}_{PSWV}({x}_{j})\cdot P({x}_{j})$$
  when *Fv*_*SRLF*_(*x*_*j*_) or *Fv*_*PSWV*_(*x*_*j*_) is less than 0.4, the fusion is performed by using the formula for *Lf*_2_(*x*_*j*_) or *Lf*_3_(*x*_*j*_), which are as follows:
  8 $$L{f}_{2}({x}_{j})=\{\begin{array}{cc}1 & fusion2 > 0.5\\ 0 & else\end{array}\,fusion2={{\rm{\beta }}}_{2}\cdot F{v}_{SLRLF}({x}_{j})\cdot P({x}_{j})$$
  9 $$L{f}_{3}({x}_{j})=\{\begin{array}{cc}1 & fusion3 > 0.5\\ 0 & else\end{array}\,fusion3={{\rm{\beta }}}_{3}\cdot F{v}_{PSWV}({x}_{j})\cdot P({x}_{j})$$
  where β_1_, β_2_, β_3_ are the fusion weight coefficients.
4. *P*(*x*_*j*_) coefficient training:

Target tissue greyscale range setting consists of the following steps:

- After the images are normalized, the pixel value ranges from 0 to 1. Divide the pixel value range 0–1 into *N*_*I*_ intervals and then extract the greyscale distribution map. Judge the target tissue distribution range based on the peak value of the greyscale map. If the number of peaks is 2 or 3, the upper boundary value of the target organization is set to $IS{N}_{\max }+Ov$, where $IS{N}_{\max }$ is the maximum interval serial number corresponding to the peak and *Ov* is the offset value.
- If the number of peaks is 1, then divide the pixel value range 0–1 into 2 × *N*_*I*_ intervals and repeat step 1.
- If the number of peaks is greater than 3, then divide the pixel value range 0–1 into *N*_*I*_/2 intervals and repeat step 1.

Establishment of the *P*(*x*_*j*_) coefficient training model.

The *P*(*x*_*j*_) coefficient training model is defined as follows:

10 $$Dsc[ds{c}_{1},ds{c}_{2},\,\cdots ds{c}_{sn}]$$

11 $$\hat{P}(x)=are\,\max \,{\Vert Dsc\Vert }_{1}\,0\le P({x}_{j})\le 1$$

where *dsc* denote the dice similarity coefficient (Dsc) of the intensity image segmentation result and the label image in an atlas, and *sn* denote the number of the atlas used for training. The *P*(*x*_*j*_) coefficient can be obtained by solving equation (11).

*P*(*x*_*j*_) coefficient training:

According to the spatial position of the target image, select the atlas images to participate in the *P*(*x*_*j*_) coefficient training. The *P*(*x*_*j*_) of the target tissue will be obtained through training and then Eq. (6) can be used to classify the target organization.

## Experiments and Results

In this section, we use the leave-one-out cross-validation method to verify the segmentation performance and robustness of the proposed method. One atlas image from the IBSR dataset serves as the target image to be segmented, and the others in the dataset are used as the atlas. The above steps are repeated until each atlas image of the IBSR dataset is segmented. In the experiments, we mainly focused on the extraction of subcortical structures. We selected six subcortical structures for segmentation, including the thalamus, hippocampus, caudate, putamen, pallidum and amygdala. We also evaluated the segmentation results of our method, the SRLF method and the PSWV method.

### Segmentation evaluation index

In order to verify the segmentation performance and robustness of the proposed method, we used the leave-one-out cross-validation method to test the proposed method. We applied four widely used segmentation metrics, Dsc, Recall, Precision and Hausdorff distance (HD) to measure the segmentation result of the proposed method. The Dsc, Recall and Precision segmentation metrics mainly measure the volume overlap ratio between the segmentation results and the manual labels, and the HD measures the surface distance between segmentation results and manual labels. These metrics are defined as follows:

12 $$Dsc(A,B)=\frac{2V(T\cap F)}{V(T)+V(F)}$$

13 $${Re}\,call(T,F)=\frac{V(T\cap F)}{V(T)}$$

14 $${\Pr }\,ecision(T,F)=\frac{V(T\cap F)}{V(F)}$$

15 $$HD(T,F)=\,{\rm{\max }}({H}_{1}(T,F),{H}_{2}(F,T))$$

16 $${H}_{1}(T,F)=\mathop{{\rm{\max }}}\limits_{{p}_{t}\in T}(\mathop{{\rm{\min }}}\limits_{{p}_{f}\in F}d({p}_{t},{p}_{f}))$$

17 $${H}_{2}(F,T)=\mathop{{\rm{\max }}}\limits_{{p}_{f}\in F}(\mathop{{\rm{\min }}}\limits_{{p}_{t}\in T}d({p}_{f},{p}_{t}))$$

where T is the target tissue pixel set in the target label image, F is the segmented target tissue pixel set, and *d*(*p*_*t*_, *p*_*f*_) is the distance between pixels p_t_ and p_f_.

All experiments and training in this study were performed on a single desktop PC (Intel(R) Core(TM) 3.70 GHz CPU).

### Target organization pixel greyscale range setting

We divide the pixel greyscale value range 0–1 into 20 intervals and determine the *P*(*x*_*j*_) of the pixels in each interval by training. In order to reduce the training time, we extracted the greyscale range of the target tissue from the greyscale distribution map. Due to the non-standard intensity between MR images, the same target tissue can have different grey distribution ranges in different images, so we determined the target tissue grey distribution range according to the grey distribution map.

**Figure 2:**
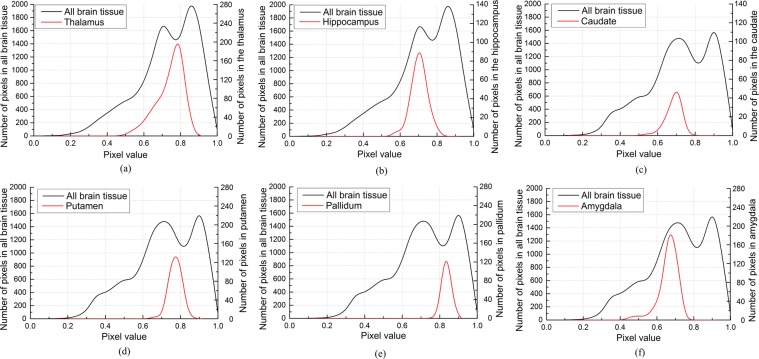
Deep brain tissue greyscale distribution map, (**a**) Thalamus, (**b**) Hippocampus, (**c**) Caudate, (**d**) Putamen, (**e**) Pallidum, (**f**) Amygdala.

As shown in Fig. 2, the pixel grey value span of deep brain tissue is between 0.3 and 0.6. We used the target tissue greyscale range-setting method mentioned above to obtain the $IS{N}_{\max }$ of the target image greyscale distribution map and then set the grey value range of the deep brain tissues according to the greyscale distribution map. The thalamus has a pixel value span of 0.6 and its maximum pixel value is $IS{N}_{\max }+0.15$; the hippocampus has a pixel value span of 0.5 and its maximum pixel value is $IS{N}_{\max }$; the caudate has a pixel value span of 0.5 and its maximum pixel value is $IS{N}_{\max }$; the putamen has a pixel value span of 0.5 and its maximum pixel value is $IS{N}_{\max }+0.1$; the pallidum has a pixel value span of 0.5 and its maximum pixel value is $IS{N}_{\max }+0.15$; and the amygdala has a pixel value span of 0.5 and its maximum pixel value is $IS{N}_{\max }-0.05$.

### Influence of the number of patches and atlas selection

It has been proven that selecting the appropriate atlas images in the MAS method can effectively improve the segmentation result^31^. The atlas images with low similarity to the target image will be discarded by atlas selection. There are large differences among slices in different positions of the human brain, so we selected atlas images to participate in target tissue segmentation based on the spatial location of the target image. Suppose the spatial position of the target image is *TSP*, *SR* is the search radius, and the images located in the space from *TSP* − *SR* to *TSP* + *SR* are selected as the images to be fused. The deep brain tissues belong to grey matter (GM) or white matter (WM). Therefore, we used GM and WM as the target tissues to test the influence of atlas selection on the segmentation results.

**Figure 3:**
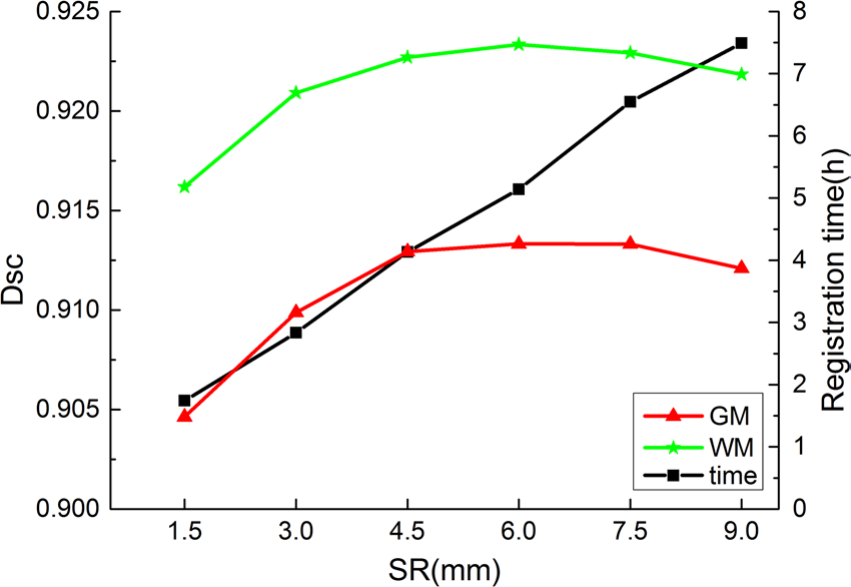
Segmentation results of different *SR*s. The green line is WM segmentation result, the red line is GM segmentation result, and the black line is the registration time.

As shown in Fig. 3, the best WM and GM Dscs were obtained when SR = 4, and the Dsc was similar to SR = 4 when SR = 3 or SR = 5. However, for every 1.5 mm increase in SR, the registration time increased by approximately 1.5 hours. Therefore, we chose SR = 3 to consider the registration time and segmentation accuracy.

Fusing the patches with lower similarity to *Tp*_*xj*_ will reduce the segmentation accuracy. In this study, we used the NCC method to measure the similarity between each *Ip*_*i*_ and *Tp*_*xj*_ and selected patches with higher similarity values for label fusion. We also tested the effect of varying the number of selected patches on the segmentation results.

**Figure 4:**
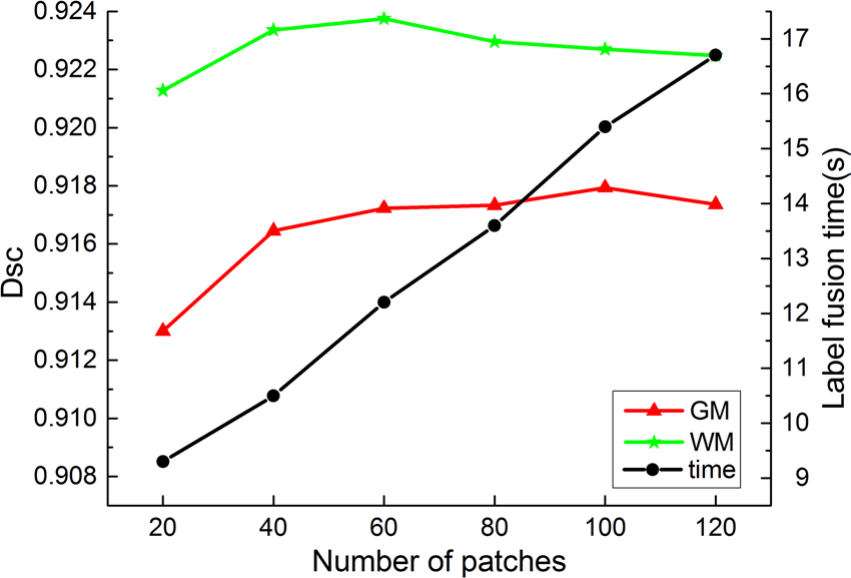
Segmentation results of numbers of selected patches. The green line is WM segmentation result, the red line is GM segmentation result, and the black line is the label fusion time.

It can be seen from Fig. 4 that the WM and GM segmentation results are best when the number of selected patches is 60, and as the number of selected patches increases, the label fusion time only increases by a few seconds. Therefore, we select 60 patches with high similarity values for label fusion.

### *P*(*x*_*j*_) coefficient training and final segmentation results

In this section, we performed segmentation tests on the thalamus, hippocampus, caudate, putamen, pallidum and amygdala. Firstly, setting the weighting factor β for each target tissue. The β_1_ is set to 3.13 for all the deep brain tissues mentioned earlier; the β_2_ = 1.25 and β_3_ = 0.67 for thalamus; the β_2_ = 1.67 and β_3_ = 2.5 for hippocampus; the β_2_ = 0.625 and β_3_ = 0.83 for caudate; the β_2_ = 0.72 and β_3_ = 1 for putamen; the β_2_ = 0.83 and β_3_ = 1.25 for pallidum; the β_2_ = 1 and β_3_ = 0.25 for amygdala. Secondly, selecting the atlas images located in *TSP* − 3 *mm* to *TSP* + 3 *mm* as the training data. Thirdly, performing segmentation training on the target organization to obtain *P*(*x*_*j*_) coefficients. Finally, using Eq. (6) to identify the target tissues.

**Figure 5:**
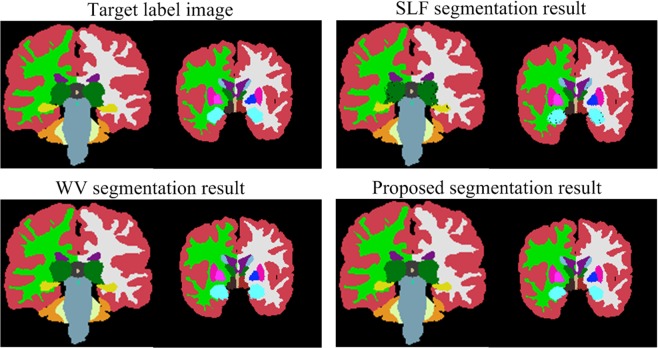
Sample segmentation results of the proposed method, SRLF method and PSWV method.

As shown in Fig. 5, there are some empty points (pixels that are not accurately classified) within the target tissues that are classified by the SRLF method, and the WV method does not work well for pixel classification of target tissue boundaries. The proposed method effectively overcomes the shortcomings of the above two methods and obtains a segmentation result similar to the target label image.

**Figure 6:**
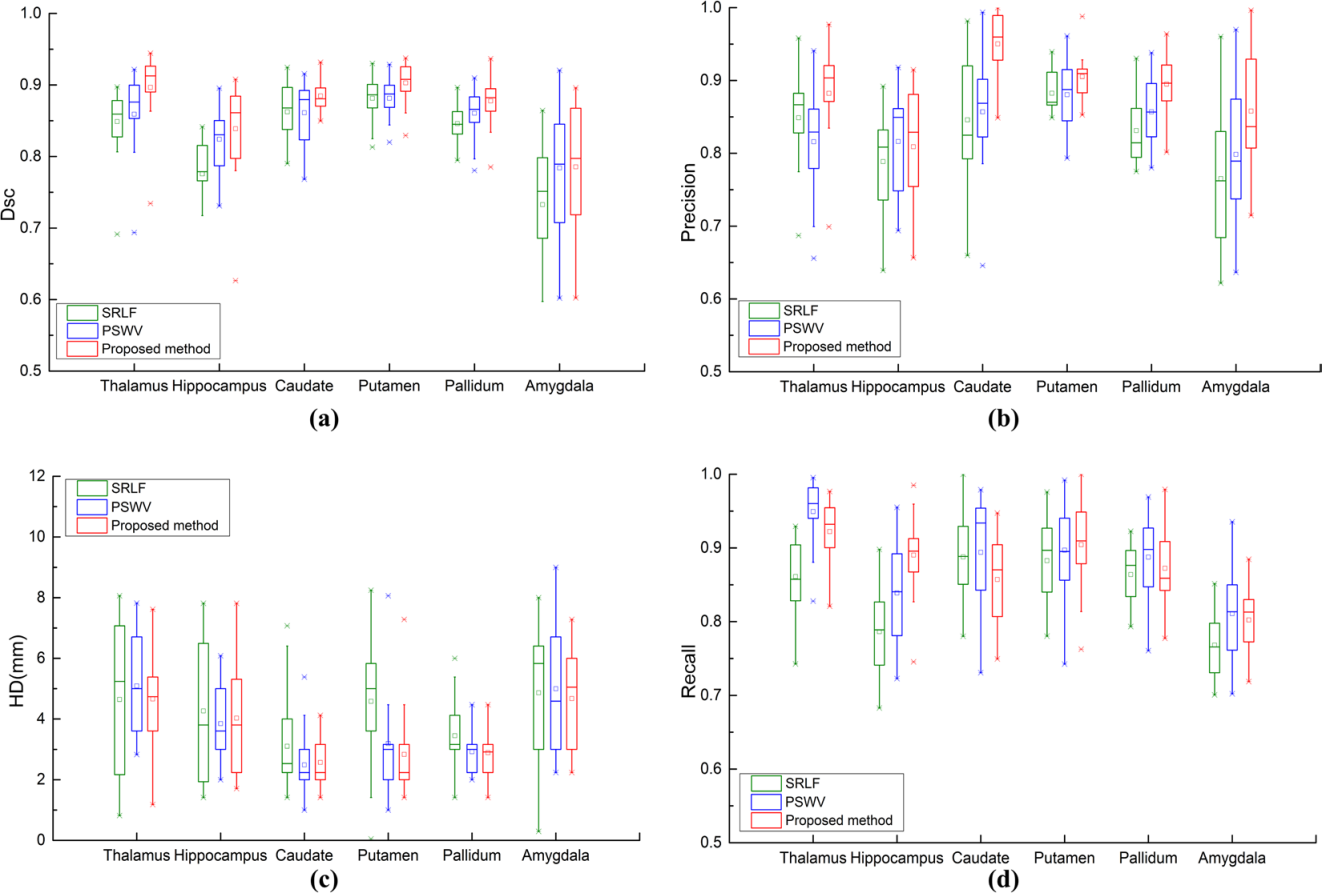
Deep brain tissue segmentation results of the proposed method, SRLF method and PSWV method.

We use four metrics to verify the segmentation performance of the proposed method, the SRLF method and the PSWV method; the segmentation results are shown in Fig. 6. It can be seen from Fig. 6(a,b) that the proposed method achieves the best Dsc and Precision results. This shows that our method achieves the best segmentation accuracy and has fewer pixels for false recognition. As shown in Fig. 6(c), for hippocampus, putamen and pallidum segmentation, the proposed method obtains the best recall results. For thalamus, caudate and amygdala segmentation, the proposed method obtains Recall results similar to those of the PSWV method. As shown in Fig. 6(d), for thalamus, caudate, putamen and pallidum segmentation, the proposed method achieves the smallest HD. For hippocampus and amygdala segmentation, the proposed method achieves an HD similar to those of the SRLF and PSWV methods.

## Discussion and Conclusion

In this section, we compare the performance of the proposed method to the related methods and state-of-the-art methods in deep brain tissue segmentation. We also introduce the limitations and future directions of the proposed method.

One disadvantage of the PSWV approach is the fusion of all extracted patches. Although the labels are assigned weights *w*_*i*_ based on patch similarity, the fusion results are still affected by the label frequency. Due to the influence of registration error, there is a large difference between the organizational boundaries of warped atlas labels and those of the target image label, which will cause the PSWV method to be unsuitable for the identification of boundary pixels. The SRLF method selects the least element from the over-complete dictionary *D*_*I*_ and represents *PT* by a linear combination of the selected elements. Therefore, most of the patches in the SRLF method are discarded, which will result in empty points in the classified target organization and reduce the segmentation accuracy.

In order to overcome the shortcomings of the above methods, We combine pixel greyscale probability information to fuse the fusion results obtained by the above two methods. Most of the segmentation errors in the multi-atlas patch-based label fusion method are concentrated on the tissue boundary. This is because the pixel gradient at the tissue boundary is not obvious, but there are still some differences in pixel grey values of different tissues. Therefore, the segmentation accuracy can be effectively improved by assigning a probability of belonging to the target tissue to different pixel values. As shown in Fig. 6, compared to the PSWV method and the SRLF method, the proposed method obtained the best Dsc and Precision results and obtained better or similar Recall and HD results.

We compared the proposed method with the state-of-the-art methods on the IBSR dataset in terms of Dsc and standard deviation. As shown in Table 1, the proposed method performed better than both FIRST and FreeSurfer for the six deep brain tissues. The overall Dsc mean for our method was significantly higher than for both of these other methods, with mean Dscs of 0.818, 0.757 and 0.872 for FISRT, FreeSurfer and the proposed method, respectively. In the pallidum and amygdala segmentation, the proposed method obtained the highest Dsc values of 0.878, 0.876, 0.801 and 0.795 for left pallidum, right pallidum, left amygdala and right amygdala, respectively. In the putamen segmentation, our method obtained the best left putamen segmentation results with a Dsc of 0.903 and obtained the same right putamen segmentation results as Xue’s method (Dsc = 0.905). The proposed method obtained similar caudate and hippocampus segmentation results to those obtained using Kaisar’s method. In the caudate segmentation, our method obtained Dscs of 0.885 and 0.898 for the left caudate and right caudate, respectively, and Kaisar’s method obtained Dscs of 0.896 and 0.896 for the left caudate and right caudate, respectively. In the hippocampus segmentation, our method obtained Dscs of 0.857 and 0.849 for the left hippocampus and right hippocampus, respectively, and Kaisar’s method obtained Dscs of 0.851 and 0.851 for the left hippocampus and right hippocampus, respectively. The proposed method obtained the third highest hippocampus segmentation accuracy among all the evaluated methods. This is because of the influence of the non-standard image intensity; the results of the thalamic segmentation of different target images using the same parameters are very different. Therefore, it is difficult to find a parameter that can fit all the images. Among the deep brain tissue segmentations mentioned in Table 1, the amygdala segmentation achieved the lowest segmentation accuracy. This is because the amygdala is relatively small in volume compared to other brain tissues and its registration effect is poor.

**Table 1:** Comparison of the proposed method with the state-of-the-art methods on IBSR dataset in terms of Dsc and standard deviation.

Target tissues	FIRST^4^	FreeSurfer^3^	Lin *et al*.^16^ method	Kaisar *et al*.^27^ method	Xu *et al*.^26^ method	Liu *et al*.^21^ method	Proposed method
L Thalamus	0.889 ± 0.018	0.830 ± 0.018	0.87 ± 0.03	0.910 ± 0.014	**0.921** ± **0.017**	0.904	0.914 ± 0.022
R Thalamus	0.890 ± 0.017	0.849 ± 0.021	0.86 ± 0.03	0.914 ± 0.016	**0.920** ± **0.018**		0.902 ± 0.024
L Hippocampus	0.809 ± 0.022	0.784 ± 0.054	0.77 ± 0.02	**0.851** ± **0.024**	0.819 ± 0.050	0.829	**0.857** ± **0.045**
R Hippocampus	0.810 ± 0.140	0.794 ± 0.025	0.76 ± 0.02	**0.851** ± **0.024**	0.842 ± 0.039		0.849 ± 0.057
L Caudate	0.797 ± 0.046	0.808 ± 0.079	0.78 ± 0.04	0.896 ± 0.018	0.824 ± 0.079	0.862	0.885 ± 0.021
R Caudate	0.837 ± 0.117	0.801 ± 0.042	0.81 ± 0.03	0.896 ± 0.020	0.854 ± 0.044		**0.898** ± **0.027**
L Putamen	0.860 ± 0.060	0.771 ± 0.039	0.84 ± 0.02	0.900 ± 0.014	0.900 ± 0.024	0.897	**0.903** ± **0.037**
R Putamen	0.876 ± 0.080	0.799 ± 0.026	0.84 ± 0.02	0.904 ± 0.012	0.905 ± 0.026		**0.905** ± **0.024**
L Pallidum	0.815 ± 0.088	0.693 ± 0.189	0.71 ± 0.07	0.825 ± 0.050	0.752 ± 0.080	0.814	**0.878** ± **0.029**
R Pallidum	0.799 ± 0.060	0.792 ± 0.085	0.74 ± 0.05	0.829 ± 0.046	0.812 ± 0.039		**0.876** ± **0.031**
L Amygdala	0.721 ± 0.053	0.585 ± 0.064	0.70 ± 0.06	0.763 ± 0.052	0.723 ± 0.076	0.753	**0.801** ± **0.055**
R Amygdala	0.707 ± 0.054	0.576 ± 0.076	0.67 ± 0.07	0.768 ± 0.058	0.729 ± 0.091		**0.795** ± **0.079**
Average	0.818	0.757	0.780	0.859	0.833	0.843	**0.872**

In the segmentation test of the deep brain tissues of the IBSR dataset, the multi-atlas registration time of each image of the proposed method was 2.83 hours. The training time of the pixel greyscale probability coefficient is 2.2–2.6 hours. The label fusion time for each image is around 15 seconds.

The limitations and future directions of the proposed method are as follows.

1. The parameter setting in the GP-LF model directly affects the quality of the segmentation result. In this study, the same target organization set the same parameter β. In fact, due to the influence of differences between images and registration errors, the fusion results of PSWV and SRLF for the same target tissue segmentation of different images are quite different. Some images have a high fusion value while some images have a low fusion value, so it is difficult to find a suitable parameter β to accommodate all subjects. A good method is to set different parameters β for different target images through a majority of segmentation training and then match the optimal parameters according to the target image information features.
2. The optimal *P*(*x*_*j*_) values differ for the same target organization of different images. In this paper, we selected the atlas images located in *TSP* − 3 *mm* to *TSP* + 3 *mm* for training to obtain adaptive *P*(*x*_*j*_), although this *P*(*x*_*j*_) is not the optimal *P*(*x*_*j*_) for each image segmentation.
3. The selection of atlas images and patches has a great impact on the segmentation results of the patch-based label fusion method. In the test, not all selected atlas images and patches were suitable; there were still atlas and patches that were not similar to the target image or to *Tp*_*xj*_, which affected the segmentation accuracy. Consequently, establishing accurate atlas and patch selection models will not only reduces segmentation time but also improves segmentation accuracy.
4. The pixels with the same grey value at the tissue boundary may belong to different tissues, which makes it difficult to classify these pixels accurately. The supervoxel-based multi-atlas segmentation method^25^ is suitable for addressing this problem and can improve the segmentation results.

## Data Availability

The datasets generated during and/or analysed during the current study are available from the corresponding author on reasonable request.
